# Missense mutation of a conserved residue in UNC-112 (kindlin) eliminates binding to PAT-4 (ILK)

**DOI:** 10.17912/micropub.biology.000454

**Published:** 2021-09-14

**Authors:** Hiroshi Qadota, Andres F Oberhauser, Guy M Benian

**Affiliations:** 1 Department of Pathology, Emory University, Atlanta, Georgia; 2 Department of Neuroscience, Cell Biology & Anatomy, Sealy Center for Structural Biology and Molecular Biophysics, University of Texas Medical Branch, Galveston, Texas

## Abstract

*C. elegans* UNC-112 (kindlin) is required for muscle sarcomere assembly, and is one component of a conserved four-protein complex that associates with the cytoplasmic tail of integrin at the base of integrin adhesion complexes in muscle. The four-protein complex consists of UNC-112 (kindlin), PAT-4 (integrin linked kinase; ILK), PAT-6 (alpha-parvin), and UNC-97 (PINCH). UNC-112 is comprised of 720 amino acid residues and contains FERM and PH domains. The N-terminal half of UNC-112 (1-396 aa) can bind to the C-terminal half of UNC-112 (397-720 aa), and this interaction is inhibited by the association of PAT-4 (ILK) to the N-terminal half of UNC-112. In support of this model, previously, we reported identification of a D382V mutation that results in lack of binding to PAT-4. However, this residue is not conserved in human Kindlins. Here, we report identification of a novel UNC-112 mutation of a conserved residue that cannot bind to PAT-4. UNC-112 E302G cannot bind to PAT-4 and does not localize to integrin adhesion complexes in muscle.

**Figure 1. A missense mutation in UNC-112 eliminates binding to PAT-4 and localization to integrin adhesion complexes in muscle:**
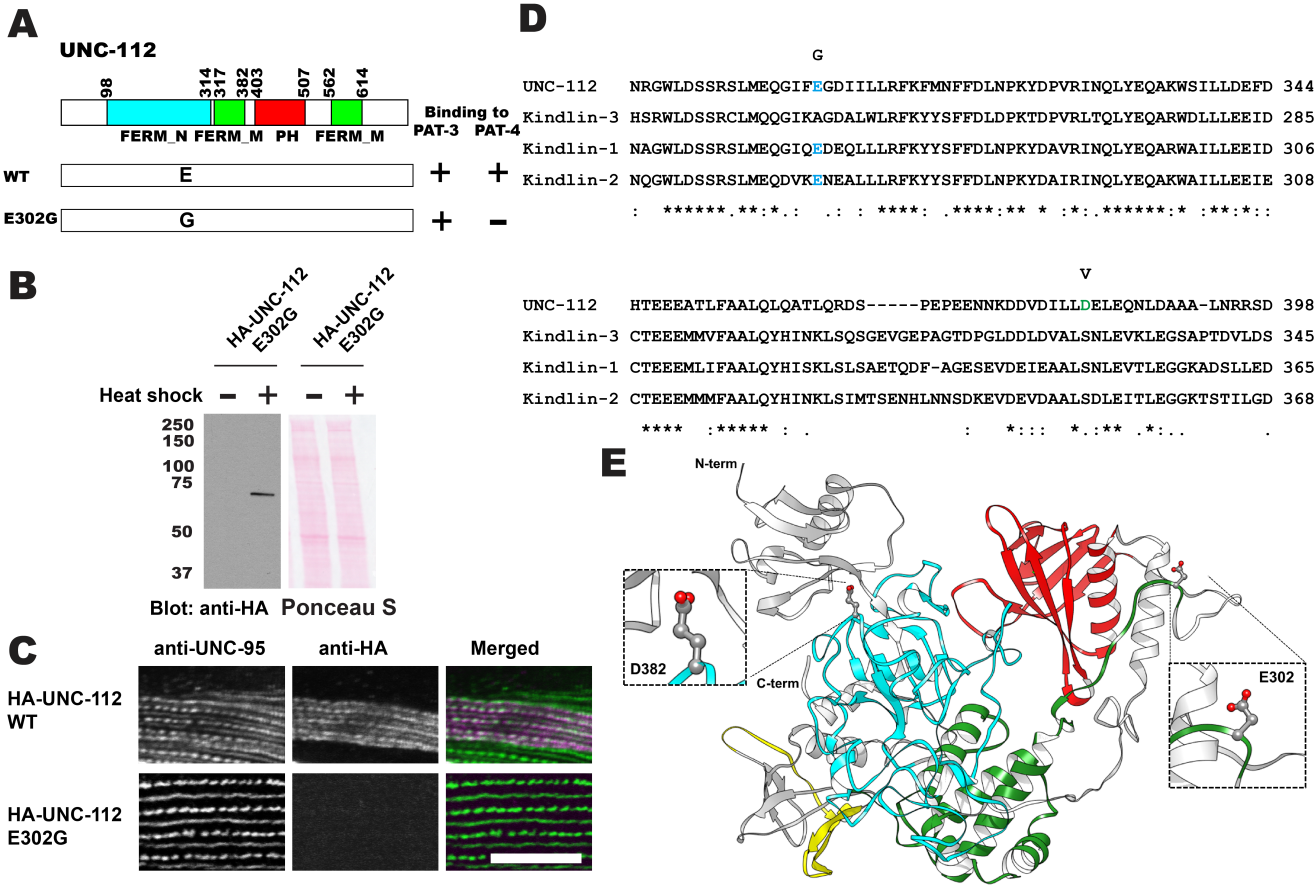
**A. A missense mutation in UNC-112 eliminates specific binding to PAT-4 using the yeast two hybrid system.** Numbers indicate amino acid residue numbers in UNC-112. + represents growth on His- plate and Ade- plate. – represents no growth on His- plate and Ade- plate. Wild type UNC-112 can bind to PAT-3 and PAT-4. UNC-112 with E302G cannot bind to PAT-4, but still can bind to PAT-3. **B. Western blot of lysates from transgenic worms.** Worms carrying HA-tagged UNC-112 E302G, expressed from a heat shock promoter, with heat shock (+) or without heat shock (−), reacted with anti-HA. **C. Localization of heat shock-expressed HA-tagged wild type and E302G UNC-112 in transgenic animals.** Worms were immunostained with anti-HA to detect the transgenic UNC-112 and with anti-UNC-95 to visualize the optical plane in body wall muscle cells that contain the integrin adhesion complexes (dense bodies and M-lines). Wild type HA-UNC-112 localizes normally to dense bodies and M-lines. However, E302G HA-UNC-112 fails to localize to these structures. White bar, 10 μm. **D. Alignment of UNC-112, Kindlin-3, Kindlin-1 and Kindlin-2.** Multiple polypeptide sequences were aligned using Clustal Omega (Madeira *et al.* 2019). E302 is shown in cyan, and D382 is shown in green. **E. Structure of UNC-112 based on the human kindlin-3 3D structure (PDB: 7C3M) (Bu *et al.*, 2020) modelled with SWISS-MODEL (Waterhouse *et al.*, 2018), and showing D382 and E302 residues.** UNC-112 with the D382V mutation also cannot bind to PAT-4 (Qadota *et al.* 2012).

## Description

*C. elegans* UNC-112 (kindlin) is required for muscle sarcomere assembly (Rogalski *et al.* 2000; Meves *et al.* 2009), and is one component of a conserved four-protein complex that associates with the cytoplasmic tail of integrin at the base of integrin adhesion complexes in muscle (Mackinnon *et al.* 2002; Lin *et al.* 2003; Norman *et al.* 2007; Qadota *et al.* 2014). UNC-112 binds directly with the cytoplasmic tail of PAT-3 (beta-integrin)(Qadota *et al.* 2012). UNC-112 binds to PAT-4 (ILK)(Mackinnon *et al.* 2002), and PAT-4 binds to both PAT-6 (alpha-parvin)(Lin *et al.* 2003), and UNC-97 (PINCH)(Mackinnon *et al.* 2002; Norman *et al.* 2007). A complex consisting of UNC-112, PAT-4, PAT-6 and UNC-97 has been demonstrated by co-immunoprecipitation (Qadota *et al.* 2014). UNC-112 is comprised of 720 amino acid residues and contains FERM and PH domains. The N-terminal half of UNC-112 (1-396 aa) can bind to the C-terminal half of UNC-112 (397-720 aa), and this interaction is inhibited by the association of PAT-4 (ILK) to the N-terminal half of UNC-112 (Qadota *et al.* 2012). In support of this model, we identified a D382V mutation that results in lack of binding to PAT-4 (Qadota *et al.* 2012). However, this residue is not conserved in human Kindlins (Figure 1D; D in UNC-112 but S in Kindlins), and mutation of this S to V in Kindlin-2 did not inhibit ILK binding (Huet-Calderwood *et al.* 2014). Here, we report identification of a novel UNC-112 mutation of a conserved residue that cannot bind to PAT-4. We found that E302G UNC-112 cannot bind to PAT-4, but still can bind to PAT-3 (beta-integrin) (Figure 1A). When we expressed HA tagged UNC-112 with E302G in *C. elegans* muscle (Figure 1B), HA tagged E302G UNC-112 cannot localize to the integrin adhesion complexes (dense bodies and M-lines) (Figure 1C). The E302 residue of UNC-112 is conserved in human Kindlin-1 and Kindlin-2, but not in Kindlin-3 (Figure 1D). Based on the only available crystal structure for a kindlin, that being for human kindlin-3 (Sun *et al.* 2020; Bu *et al.* 2020), we generated a homology model of UNC-112 and highlighted the locations of E302 and D382 (Figure 1E). Both E302 and D382 are located on surface loops of the structure. Mutating E302 to G, or mutating D382 to V resulted in no clashes with neighboring residues, based on the rotamer mutagenesis and energy minimization tools in Chimera (Pettersen *et al.*, 2004). In fact, the lack of a sidechain of G, or the smaller sidechain of V, is likely to make these loops even more flexible. These mutations are predicted to not alter the overall structure of UNC-112 but could possibly affect the surface binding of UNC-112 to PAT-4. It should be noted, that in addition to our findings, a L located 6 residues C-terminal of D382, within the same FERM_M domain, and conserved in UNC-112 and all human kindlins (Figure 1D), when mutated to A, also greatly reduces binding to ILK (Huet-Calderwood *et al.* 2014). However, the results by Huet-Calderwood *et al.* were obtained by GST pulldown from lysates of tissue culture cells that overexpress both ILK and alpha-parvin. Our results, using the yeast two hybrid method and localization in worm muscle cells in which only UNC-112 was overexpressed, provide more evidence for an effect on direct binding between UNC-112 (kindlin) and PAT-4 (ILK). Our finding of a conserved residue in UNC-112 in the FERM_N domain that reduces binding to PAT-4, opens the door to testing the comparable residues in human Kindlin-1 and Kindlin-2 for their importance to binding to ILK. We do not know what the phenotype is of a nematode that is homozygous for the UNC-112 E302G mutation. Since UNC-112 function requires PAT-4 (Mackinnon *et al.* 2002), and all known mutations in *pat-4* are Pat embryonic lethal, worms homozygous for UNC-112 E302G, might be Pat embryonic lethal. Several mutations in *unc-112* are known to result in either the Pat embryonic phenotype or the adult viable Unc phenotype in which adults move slowly and have a disorganized myofilament lattice (Rogalski *et al.* 2000). The three known Pat alleles are *unc-112(st562)* and *unc-112(st581)* that are both nonsense mutations, and *unc-112(gk1)* that is a deletion. The Unc allele, *unc-112(r367)*, is a missense mutation, T85I, and is also temperature sensitive. The molecular properties of UNC-112 T85I have not been explored, and thus, we do not know if the phenotype is due to reduced binding to PAT-4 or PAT-3, or whether it results in a unstable protein. Finally, there is one unusual allele created by CRISPR/Cas9, *unc-112(kq715)*, L715E, which shows a defect in the migration of the distal tip cell (Park *et al.* 2020), but whether there was an effect on the myofilament lattice of body wall muscle, was not reported.

## Methods

**Yeast two-hybrid screening**

Random mutagenesis using PCR and screening for interactions using the yeast two hybrid method was performed as previously described (Miller *et al.* 2006; Qadota *et al.* 2012). The UNC-112 N-terminal half was cloned into pGAD-C3 (prey plasmid), and the UNC-112 C-terminal half was cloned into pGBDU-C1 (bait plasmid). PAT-4 full length cDNA was cloned into pGBDU-C2 (bait plasmid). The N-terminal UNC-112 fragment was amplified by the error-prone method using the following primers: 5′ primer, AAA AAA GAG ATC GAA TTC CCC GGG GGA TCC; 3′ primer, GGT TTT TCA GTA TCT ACG ATT CAT AGA TCT. These primers were designed to amplify the insert, and each consists of 30 nucleotides of pGAD-C3. The cloning of error-prone PCR-amplified fragments into the acceptor plasmid was accomplished by exploiting yeast recombination in vivo. The mixture of the amplified PCR fragments (∼1 μg) and the acceptor plasmid (1 μg) digested with BamHI and BglII was transformed into PJ69-4A harboring pGBDU-UNC-112C. Transformed yeast cells were spread onto −Leu−Ura−His and 2 mM 3-amino-1,2,4-triazole to screen for His+ colonies. His+ selection ensured that the mutagenized UNC-112N could still interact with UNC-112C. This step was essential for eliminating clones with premature stop mutations or with many other mutations. His+ colonies were streaked onto an −Ade plate and screened for His+Ade+ colonies. After streaking on a 5-fluoroorotic acid plate to eliminate the URA3 marker bait plasmid (pGBDU-UNC-112C), prey clones were isolated from yeast and amplified in *E. coli*. From His+Ade+ yeast colonies, 64 mutagenized clones were isolated. These prey clones were transformed separately into PJ69-4A carrying either pGBDU-UNC-112C or pGBDU-PAT-4 (full-length) to check for interaction with the C-terminal half of UNC-112 and interaction with full-length PAT-4. Among 64 mutagenized clones of UNC-112N, 8 of these clones could not bind to PAT-4. From DNA sequencing of these 8 clones, we identified one clone with a single amino acid change, E302G. The UNC-112N with E302G was cloned into pACT-Q-UNC-112C (Qadota *et al.* 2012), resulting in pACT-Q-UNC-112 E302G (full length), and then used to test for binding to PAT-3.

**Expression in *C. elegans***

To express HA-UNC-112 E302G in *C. elegans* using a heat shock promoter, the SmaI-EcoRV fragment of pACT-Q-UNC-112 E302G was inserted into EcoRV digested pKS-HA-UNC-112-Acp (Qadota *et al.* 2012), resulting in pKS-HA-UNC-112 E302G. The NheI fragment of pKS-HA-UNC-112 E302G was then cloned into NheI-digested pPD49.78 and pPD49.83, resulting in pPD49.78-HA-UNC-112 E302G and pPD49.83-HA-UNC-112 E302G. pPD49.78/83-HA-UNC-112 E302G were mixed with pTG96 (SUR-5::NLS::GFP) (Yochem *et al.*, 1998)as a transformation marker and injected into wild type N2 worms. Transgenic lines with extrachromosomal arrays containing pPD49.78/83-HA-UNC-112 E302G and pTG96 (GB339; sfEx74 [sur-5::GFP; hspp::HA::unc-112 E302G]) were established by picking GFP-positive worms using a GFP dissection microscope. Generation and characterization of transgenic animals expressing by heat shock the comparable HA-tagged wild type UNC-112 was described previously (Qadota *et al.* 2012). Expression of the HA-tagged UNC-112 proteins (E302G and wild type) was induced by incubation of the transgenic worms at 30 °C for 2 h (heat shock).

**Fluorescence imaging**

Heat-shocked transgenic worms were fixed (Nonet *et al.* 1993)and stained by anti-GFP (to verify the existence of extrachromosomal array; rabbit polyclonal from Thermo Fisher A11122), anti-UNC-95 (to identify dense bodies and M-lines in muscle cells; rabbit polyclonal Benian-13, Qadota *et al.* 2007), and anti-HA (to determine the localization of HA-tagged UNC-112 proteins; Sigma-Aldrich H3663; 1:200 dilution). Images were captured at room temperature with a Zeiss confocal system (LSM510) equipped with an Axiovert 100M microscope and an Apochromat ×63/1.4 numerical aperture oil objective, in ×2.5 zoom mode. The color balances of the images were adjusted by using Adobe Photoshop version 22.4.3.

**Western blotting**

We prepared worm lysates (Hannak *et al.* 2002) from transgenic worms with or without heat shock and examined the expression of HA-tagged UNC-112 proteins by Western blot, reacting with anti-HA (Sigma-Aldrich H3663; 1:200 dilution).

**Protein structure modeling**

For UNC-112 protein structure modeling, CLUSTALW version 1.2.2 (https://www.ebi.ac.uk/Tools/msa/clustalw2/), SWISS-MODEL version July 2021 (https://swissmodel.expasy.org/; Waterhouse *et al.*, 2018) and Phyre2 version 2.0 (http://www.sbg.bio.ic.ac.uk.phyre2/html/page.cgi?id=index; Kelley LA *et al.*, 2015) online tools were used. Human kindlin-3 (7C3M.pdb; Bu *et al.* 2020) was used as reference crystal structure. Molecular graphics were generated by using Chimera version 1.15 (https://www.cgl.ucsf.edu/chimera/; Pettersen *et al.*, 2004).
